# Comparative Prognostic Performance of Nutritional Indices in Acute Myeloid Leukemia

**DOI:** 10.3390/jcm15166179

**Published:** 2026-08-10

**Authors:** Gokhan Burul, Hasan Göze, Isa Yalcınkaya, Tahir Alper Cinli, Mesut Ayer, İstemi Serin

**Affiliations:** Department of Hematology, Başakşehir Çam and Sakura City Hospital, 34480 Istanbul, Turkey; hasangoze@gmail.com (H.G.); drisayalcinkaya@gmail.com (I.Y.); tahiralper@gmail.com (T.A.C.); mesutayerdr@hotmail.com (M.A.); serinistemi@hotmail.com (İ.S.)

**Keywords:** acute myeloid leukemia, Nutritional Risk Index, CONUT, Prognostic Nutritional Index, overall survival, prognosis

## Abstract

**Background/Objectives**: Malnutrition adversely affects the clinical course of acute myeloid leukemia (AML), yet comparative data on objective nutritional indices remain limited. **Methods**: This study evaluated the prognostic performance of four indices—Controlling Nutritional Status (CONUT), Simple CONUT, Prognostic Nutritional Index (PNI), and Nutritional Risk Index (NRI)—on overall survival (OS) alongside the European LeukemiaNet (ELN) 2022 risk classification. Pre-treatment parameters of 236 newly diagnosed AML patients were retrospectively analyzed using Receiver Operating Characteristic (ROC) curves and multivariate Cox regression models. **Results**: In ROC analysis, only NRI demonstrated significant discriminative performance in predicting OS (Area Under the Curve = 0.637, *p* < 0.001), whereas CONUT, Simple CONUT, and PNI lacked statistical significance. Patients with low NRI scores had significantly worse survival than the high NRI group (median OS: 16.8 months versus not reached, *p* = 0.004), particularly within the ELN favorable risk category (2-year OS: 59.6% versus 93.5%, *p* = 0.022). Multivariate analysis established NRI as an independent predictor of mortality (Hazard Ratio = 0.967, *p* = 0.009) alongside age and ELN risk. **Conclusions**: In conclusion, NRI outperforms other nutritional indices and serves as an independent prognostic factor in AML, offering a more holistic and effective approach to clinical risk stratification.

## 1. Introduction

Malnutrition adversely affects the clinical course of cancer patients by increasing the risk of complications and leading to a significant decline in overall quality of life [1]. Hematological malignancies require clinical management strategies that differ from those for solid tumors because of their aggressive biological characteristics and complex treatment courses [2]. In these malignancies, inflammation- and catabolism-driven processes make nutritional status even more critical [3]. Acute myeloid leukemia (AML) is an aggressive hematological malignancy characterized by the clonal proliferation of hematopoietic precursor cells, which may lead to rapid clinical deterioration and requires prompt treatment [4]. In AML, treatment selection and prognostic assessment are largely based on the genetic risk stratification recommended by the European LeukemiaNet (ELN) 2022 guidelines [5]. However, this classification primarily reflects the biological characteristics of the disease and does not directly account for host-related factors such as performance status, systemic inflammation, and nutritional reserves [5].

Systemic inflammation, increased catabolic activity, and treatment-related complications that may develop during the clinical course of AML can contribute to the deterioration of patients’ nutritional status [6]. Although comprehensive nutritional screening tools, such as the Nutritional Risk Screening 2002 (NRS-2002) and the Patient-Generated Subjective Global Assessment (PG-SGA), are valuable for assessing nutritional status, these methods rely on patient history, clinical evaluation, and qualitative components [1]. In patients with AML, the dynamic course and clinical heterogeneity of the disease make standardized and reliable assessment of nutritional status challenging [1,6]. Therefore, objective nutritional indices have attracted increasing interest because of their potential prognostic value. In recent years, nutritional indices based on serum albumin, lymphocyte count, and body weight, including the Controlling Nutritional Status (CONUT) score, Prognostic Nutritional Index (PNI), and Nutritional Risk Index (NRI), have emerged as practical and easily calculated tools for the simultaneous assessment of nutritional and immune status in patients with malignancies [7,8,9]. By reflecting the combined effects of inflammation and malnutrition, these indices have been shown to provide valuable prognostic information across various malignancies [10]. Furthermore, accumulating evidence suggests that these indices may be associated with treatment response and survival in patients with hematological malignancies [11,12,13]. Nevertheless, studies simultaneously evaluating genetic risk stratification and nutritional status in patients with AML remain limited [14].

The CONUT score is an objective laboratory-based index that evaluates nutritional status using serum albumin concentration, total lymphocyte count, and total cholesterol level [7]. By integrating parameters related to protein reserves, immune status, and metabolic status into a single score, it provides a rapid and reproducible assessment. However, because serum albumin and total lymphocyte count can be influenced by systemic inflammation, infection, and disease activity, the results should be interpreted with caution [10]. The simplified CONUT (sCONUT) score was developed by excluding the lymphocyte component and is based solely on serum albumin and total cholesterol levels to overcome the limitations associated with total lymphocyte count, which may be affected by disease burden, bone marrow failure, infection, and inflammation in hematological malignancies such as AML [14]. The PNI combines serum albumin concentration and total lymphocyte count to provide information on a patient’s nutritional reserves and immune status [15]. Owing to its simple calculation and reliance on routinely available laboratory parameters, it has been investigated as a prognostic marker in various solid tumors and hematological malignancies [8,16]. However, the components of the PNI may also be influenced by systemic inflammation, disease activity, and treatment-related changes [10].

The NRI is an index that assesses nutritional risk using serum albumin concentration together with the ratio of current body weight to ideal body weight [9]. By incorporating body weight changes, it contributes to the assessment of protein–energy status; however, changes in body weight caused by edema and alterations in fluid balance represent important limitations of the index [11]. The present study aimed to compare the prognostic performance of four nutritional indices (CONUT, sCONUT, PNI, and NRI) for overall survival (OS) in patients with AML based on the ELN genetic risk classification. Although these indices share several common components, they reflect different aspects of nutritional status, including immune status, protein reserves, metabolic status, and changes in body weight. Therefore, by simultaneously analyzing the CONUT, sCONUT, PNI, and NRI at the time of diagnosis, we sought to directly compare their prognostic performance and clinical applicability in patients with AML. In addition, we aimed to determine whether these nutritional indices provide incremental prognostic value beyond the ELN 2022 genetic risk classification.

## 2. Materials and Methods

### 2.1. Study Population

In this retrospective cohort study, a total of 236 newly diagnosed patients with AML at our tertiary hospital’s adult hematology unit between January 2021 and June 2025 were included. Patients younger than 18 years of age and those previously diagnosed with or treated for AML were excluded from the study. Patients with missing laboratory data (serum albumin, total cholesterol, and total lymphocyte count) or anthropometric data (body weight and height) required for the calculation of CONUT, sCONUT, PNI, and NRI were excluded. Patients’ demographic characteristics (age, sex), height and weight measurements, laboratory parameters (white blood cell count, hemoglobin, platelet count, lymphocyte count, serum albumin, and total cholesterol levels), cytogenetic and molecular features, administered treatment protocols, and treatment responses were evaluated. Patients were stratified into favorable, intermediate, and adverse risk groups according to the ELN risk classification [5]. Table 1 summarizes the cytogenetic and molecular features used for the ELN 2022 genetic risk classification [5].

### 2.2. Treatment Strategies

Treatment strategies were determined according to patients’ clinical characteristics, performance status, and eligibility for intensive chemotherapy (IC) [5]. Patients considered eligible for IC received standard 7 + 3 induction chemotherapy consisting of an anthracycline and cytarabine, whereas older patients or those deemed unfit for IC received low-intensity therapy with a hypomethylating agent in combination with venetoclax. Patients who achieved CR subsequently received consolidation therapy, and selected patients underwent allogeneic hematopoietic stem cell transplantation (HSCT) according to their ELN risk stratification [5].

### 2.3. Treatment Response Definitions

Treatment responses were assessed using bone marrow biopsy findings and peripheral blood counts according to the recommendations of the European Hematology Association (EHA) and ELN expert panel [17]. Complete remission (CR) was defined as the presence of <5% blasts in the bone marrow, an absolute neutrophil count ≥1000/µL, and a platelet count ≥100,000/µL. For treatment response analyses, patients were classified into two groups: CR and non-CR. The non-CR group included all patients who failed to achieve complete remission after induction therapy, including those with partial remission or refractory disease.

### 2.4. Nutritional Risk Indices

The CONUT score was calculated using serum albumin (g/dL), total cholesterol (mg/dL), and total lymphocyte count (cells/µL), as detailed in Table 2A [7]. Additionally, in line with previous reports, the “Simple CONUT” score was calculated by excluding the total lymphocyte count from the evaluation criteria, as shown in Table 2B [14].

The NRI was calculated using the following formula: NRI = (1.519 × serum albumin, g/L) + (41.7 × current body weight, kg/ideal body weight, kg).

The ideal body weight (IBW) was determined using the Lorentz formula:

- For men: IBW = (height in cm − 100) − ((height in cm − 150)/4).
- For women: IBW = (height in cm − 100) − ((height in cm − 150)/2).

An NRI score of 100 or higher was classified as normal, 97.5–99.9 as mild malnutrition, 83.5–97.4 as moderate malnutrition, and less than 83.5 as severe malnutrition [9].

The PNI was calculated using the following formula: PNI = 10 × serum albumin (g/dL) + 0.005 × lymphocyte count (cells/mm^3^).

A PNI score of 45 or higher was considered normal nutritional status, whereas a score of less than 45 was defined as malnutrition [15]. Height and body weight measurements for all patients were recorded on the day of hospital admission.

### 2.5. Statistical Analysis

Statistical analyses of the research data were performed using the SPSS version 26.0 software package (IBM Corp., Armonk, NY, USA). Descriptive statistics for continuous variables were presented as mean, standard deviation, median, minimum, and maximum values, whereas categorical variables were expressed as frequencies (*n*) and percentages (%). OS was calculated from the date of diagnosis to the date of death or the last follow-up. To determine the OS durations and survival probabilities of the patients, the Kaplan–Meier survival analysis was utilized, and the statistical significance of survival differences between groups was evaluated using the log-rank test. Receiver Operating Characteristic (ROC) curve analysis was applied to determine the discriminative performance and optimal cut-off values of the nutritional indices (CONUT, Simple CONUT, PNI, and NRI) in predicting survival status. The Area Under the Curve (AUC) values, 95% Confidence Intervals (CIs), sensitivity, and specificity calculated from this analysis were reported. To identify the prognostic factors influencing OS, univariate and multivariate Cox proportional hazard regression analyses were performed. The results of the analysis were presented as Hazard Ratios (HRs) and 95% CIs. For all analyses, a two-sided *p*-value of <0.05 was considered statistically significant.

### 2.6. Ethical Statement

The study was conducted in accordance with the ethical principles described in the Declaration of Helsinki. Institutional review board and ethics committee approval for this study was obtained from the Ethics Committee of Basaksehir Cam and Sakura City Hospital (protocol no: 158, date of approval: 4 March 2026). Due to the retrospective nature of the study and the use of completely anonymized patient data, the requirement for informed consent was formally waived by the Ethics Committee of Basaksehir Cam and Sakura City Hospital.

## 3. Results

### 3.1. Patient Characteristics

Baseline demographic and clinical characteristics are summarized in Table 3. The median age of the cohort was 55 years (range, 18–85). Of the patients, 59.3% (*n* = 140) were male and 40.7% (*n* = 96) were female. Regarding treatment intensity, 63.1% (*n* = 149) of the patients received intensive therapy, while 36.9% (*n* = 87) received low-intensity treatment. In terms of treatment response, 77.1% (*n* = 182) of the patients achieved complete remission (CR), whereas 22.9% (*n* = 54) were in the non-CR group. According to the ELN 2022 risk classification, 53% (*n* = 125) of the patients were categorized into the adverse risk group, 19.1% (*n* = 45) into the intermediate risk group, and 28% (*n* = 66) into the favorable risk group. The median follow-up duration was 25.2 months. At the time of the last follow-up, 61.9% (*n* = 146) of the patients were alive, while 38.1% (*n* = 90) had died. For the entire cohort, the 2-year and 3-year OS rates were calculated as 51% and 42.5%, respectively.

Pre-treatment anthropometric measurements and laboratory data of the patients are presented in Table 4. In terms of physical measurements, the mean height was 167.77 ± 9.22 cm, the mean body weight was 76.22 ± 14.63 kg, and the mean body mass index (BMI) was 27.13 ± 5.12 kg/m^2^. Regarding laboratory parameters, the median white blood cell count was 10,210 (range, 400–322,500)/µL, the mean hemoglobin level was 8.0 ± 1.94 g/dL, the median platelet count was 37 (range, 4–550) × 10^3^/µL, and the median lymphocyte count was 2550 (range, 70–222,300)/µL. Among biochemical parameters, the mean total cholesterol level was found to be 140.43 ± 42.94 mg/dL, and the mean serum albumin level was 3.86 ± 0.55 g/dL.

### 3.2. Nutritional Status and Index Scores

The distribution of patients’ nutritional status and their respective index scores are detailed in Table 5. According to the CONUT score, 24.6% of the patients were classified as normal, 55.5% as mild, 18.6% as moderate, and 1.3% as severe malnutrition. Based on the Simple CONUT scoring system, 41.1% of the patients were evaluated as normal, 40.7% as mild, 13.6% as moderate, and 4.7% as severe malnutrition, respectively. In terms of PNI, while 75.8% of the patients were within normal limits, 24.2% were in the low-risk group. When the distribution according to the NRI was analyzed, it was found that 53.4% of the patients were in the normal group, 10.2% in the mild risk group, 32.2% in the moderate risk group, and 4.2% in the severe risk group. The mean CONUT score was determined as 2.92 ± 2.00, the Simple CONUT score as 2.12 ± 1.71, the PNI value as 80.30 ± 95.17, and the NRI value as 99.80 ± 8.87.

### 3.3. ROC Analysis and Optimal Cut-Off Values

**Figure 1:**
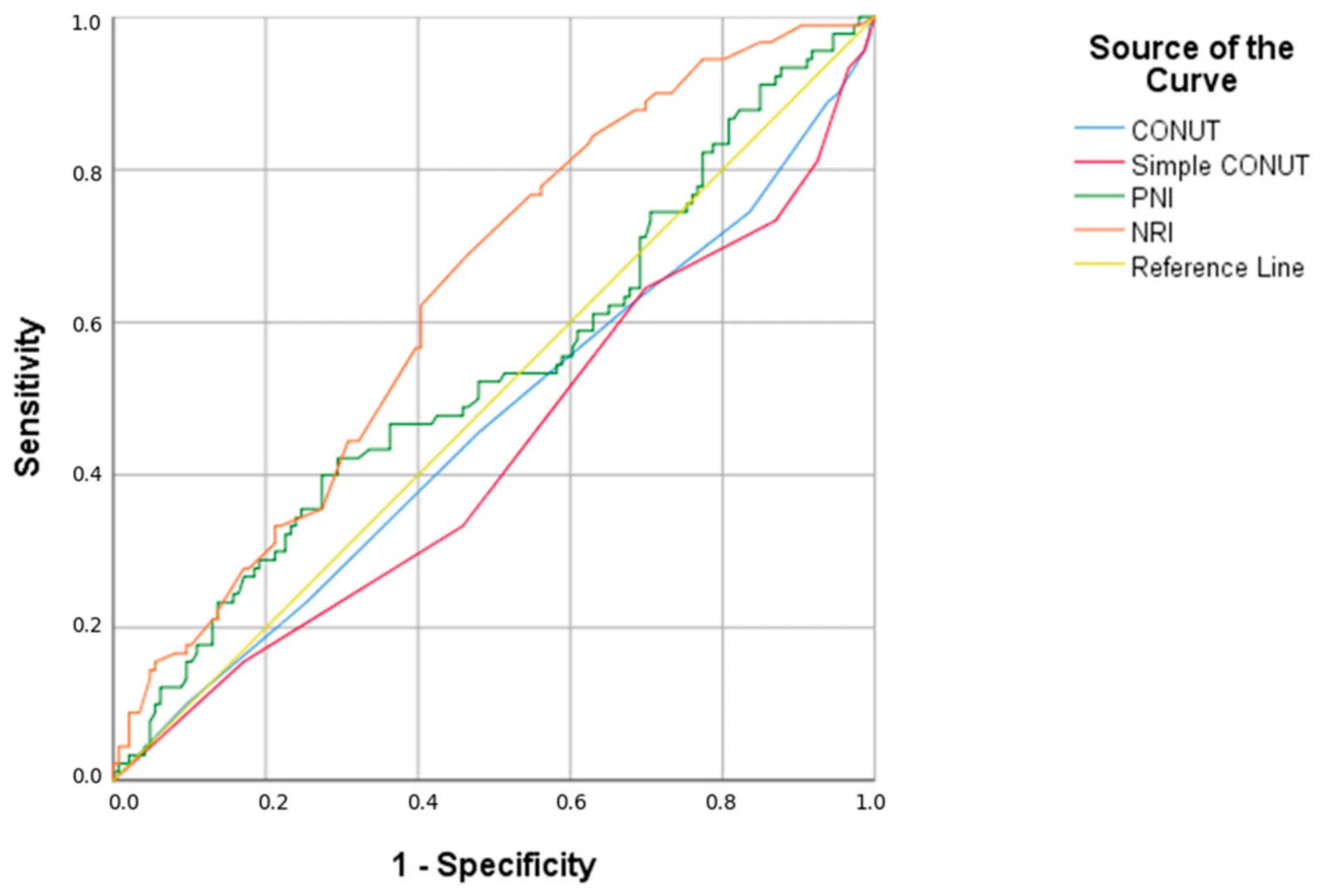
Comparison of ROC curves for the four nutritional indices.

The prognostic performance of the nutritional indices is displayed in Table 6, and the corresponding ROC curves are presented in Figure 1. In the ROC curve analysis, NRI demonstrated a significant and moderate discriminative performance in predicting OS (AUC = 0.637; 95% CI: 0.566–0.707; *p* < 0.001). Using the Youden index, the optimal cut-off value for NRI was determined to be 101.70, yielding a sensitivity of 69% and a specificity of 53%. In contrast, the scores of CONUT (AUC = 0.465; *p* = 0.366), Simple CONUT (AUC = 0.430; *p* = 0.070), and PNI (AUC = 0.537; *p* = 0.340) were not found to be statistically significant in predicting survival.

### 3.4. Kaplan–Meier Survival Analysis

**Figure 2:**
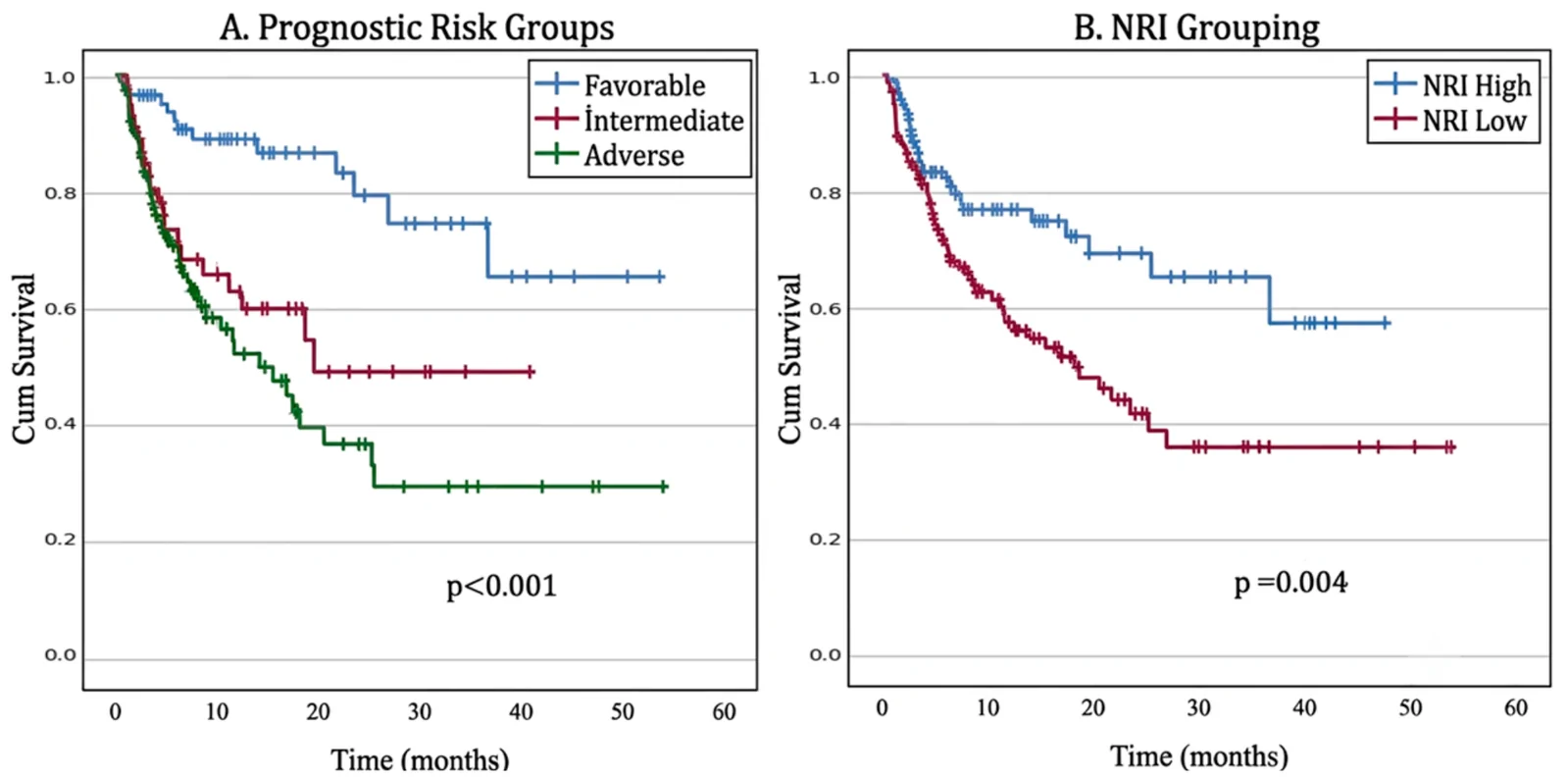
Kaplan–Meier overall survival curves according to (**A**) the ELN risk classification and (**B**) the Nutritional Risk Index (NRI). For NRI analysis, patients are stratified into high-NRI (≥101.70) and low-NRI (<101.70) groups.

As shown in Table 7, OS was significantly poorer in patients with a low NRI score (<101.70) compared with those in the high NRI group (≥101.70) (*p* = 0.004; Figure 2B). The 2-year OS rate was 67.0% in the high-NRI group and 40.1% in the low-NRI group. Median OS was not reached in the high-NRI group, whereas it was 16.8 months in the low-NRI group. The ELN risk classification also effectively stratified patients according to OS (*p* < 0.001; Figure 2A). Details of the numbers of patients at risk are provided in Supplementary Table S1.

### 3.5. Prognostic Value of the NRI Score

**Figure 3:**
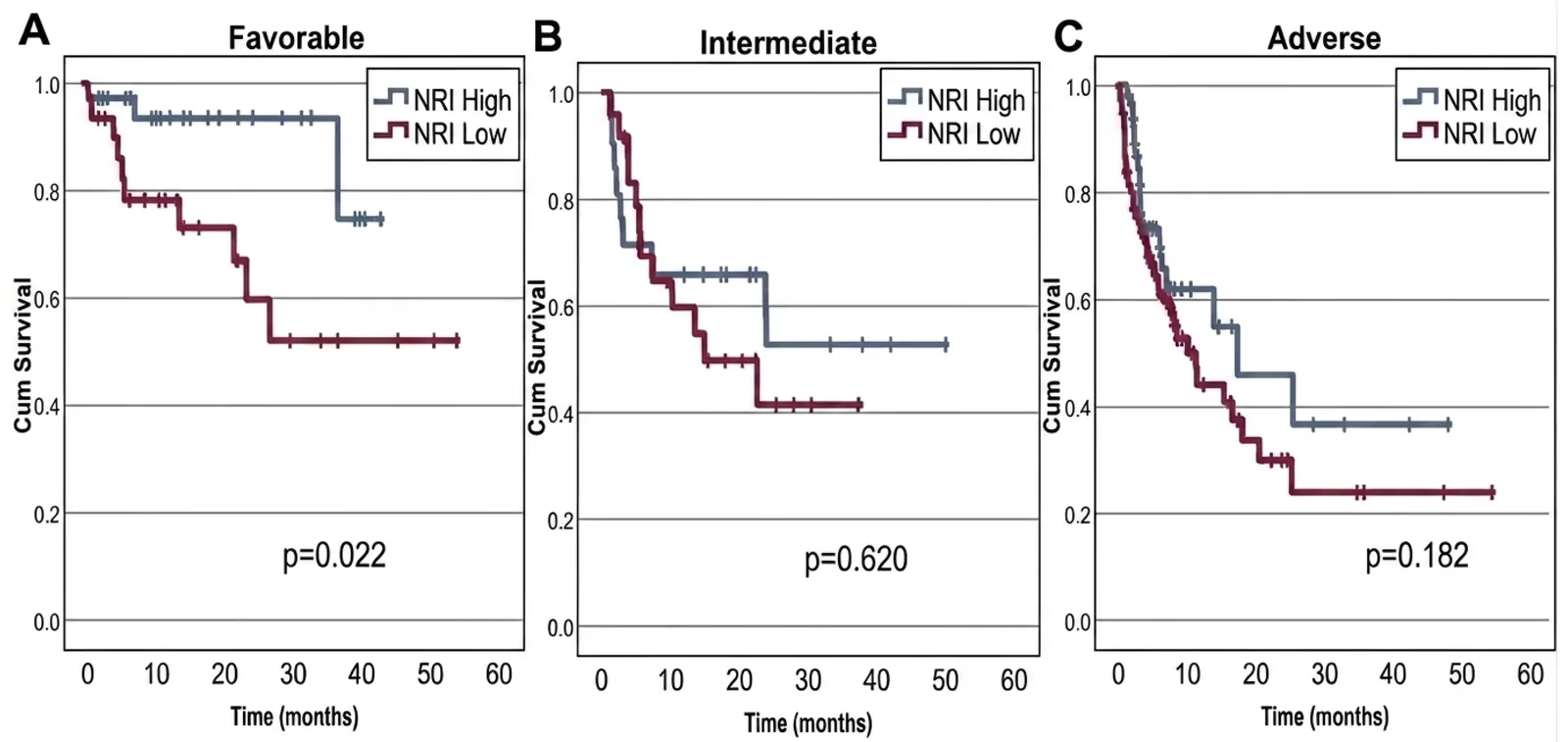
Kaplan–Meier curves for overall survival according to NRI score across ELN risk groups: favorable risk (**A**), intermediate risk (**B**), and adverse risk (**C**). In each subgroup, patients are stratified into high-NRI (≥101.70) and low-NRI (<101.70) groups.

In Table 8, we evaluated whether the NRI score could provide a more detailed risk stratification within the ELN risk groups. While no significant prognostic impact of the NRI score was observed within the adverse and intermediate risk groups (Figure 3B,C), a significantly worse survival was observed in patients with a low NRI score compared to the high NRI group within the favorable risk category (2-year OS: 59.6% vs. 93.5%; *p* = 0.022; Figure 3A). In terms of median OS, the median was not reached in the NRI-high arm within the favorable and intermediate risk groups, whereas it was found to be 17.43 and 10.33 months for the NRI-high and NRI-low groups within the adverse risk category, respectively.

### 3.6. Univariate and Multivariate Cox Regression Analysis for Survival Time

The results of the univariate Cox regression analysis evaluating the prognostic factors for overall survival are presented in Table 9. In this analysis, patients in the ELN intermediate and adverse risk categories demonstrated a significantly increased risk of mortality compared to those in the favorable risk group (HR = 2.768, *p* = 0.004 and HR = 3.758, *p* < 0.001, respectively). Advanced age (HR = 1.023, *p* < 0.001) and a higher Simple CONUT score (HR = 1.143, *p* = 0.023) were also significantly associated with increased mortality risk. Conversely, an increase in NRI scores was found to significantly reduce the risk of mortality (HR = 0.960, 95% CI: 0.937–0.983, *p* = 0.001).

To further evaluate these findings, two separate multivariate Cox regression models were constructed for OS, as detailed in Table 10. In Model A (the multivariate model including Simple CONUT), a significantly increased mortality risk was observed in the ELN intermediate (HR = 2.265, 95% CI: 1.104–4.652, *p* = 0.026) and adverse risk (HR = 3.077, 95% CI: 1.641–5.771, *p* < 0.001) groups. Additionally, advanced age (HR = 1.016, 95% CI: 1.003–1.029, *p* = 0.015) and the Simple CONUT score (HR = 1.135, 95% CI: 1.018–1.284, *p* = 0.035) were identified as independent prognostic factors for OS.

In Model B (the multivariate model including NRI), ELN intermediate risk (HR = 2.101, 95% CI: 1.015–4.349, *p* = 0.046), ELN adverse risk (HR = 2.851, 95% CI: 1.513–5.371, *p* = 0.001), and advanced age (HR = 1.015, 95% CI: 1.002–1.028, *p* = 0.020) maintained their independent prognostic significance. Furthermore, NRI remained statistically significant in the multivariate analysis (HR = 0.967, 95% CI: 0.942–0.992, *p* = 0.009), establishing itself as a significant predictor of mortality independent of ELN risk categories and age.

## 4. Discussion

Nutritional status influences prognosis through multiple pathways; malnutrition increases treatment-related toxicity and length of hospital stay while reducing quality of life. The prognostic impact of nutritional status in hematological malignancies is more heterogeneous and complex compared with solid tumors [1]. In diseases such as leukemia and lymphoma, inflammation, bone marrow dysfunction, and increased catabolic activity may alter albumin levels, total lymphocyte counts, and lipid metabolism [10]. Therefore, NRI, CONUT, sCONUT, and PNI may reflect not only nutritional status but also disease-related inflammatory processes [7,8,9]. In our study, NRI demonstrated superior prognostic performance compared with CONUT, sCONUT, and PNI. This difference may be related to the extent to which the biological components included in these indices are affected by the pathophysiology of AML. The lymphocyte count included in PNI and CONUT scores may also reflect leukemic burden, bone marrow suppression, and treatment-related factors in AML. Similarly, total cholesterol, a component of CONUT, may be influenced by cancer-related systemic inflammation and metabolic alterations [10]. In contrast, NRI integrates serum albumin and body weight, potentially providing a more comprehensive reflection of changes in patients’ nutritional reserves and physiological status [9].

The prognostic value of PNI as an independent predictor has been demonstrated in solid malignancies, with low PNI levels being associated with poor survival outcomes [15]. Its role in hematological malignancies has been increasingly investigated in recent years [18,19]. Begendi et al. demonstrated that low PNI was an independent prognostic factor for OS in patients with newly diagnosed multiple myeloma (AUC: 0.667; HR: 2.84) [20]. Tang et al. showed that low pre-transplant PNI levels were independently associated with 180-day mortality in AML patients undergoing allogeneic HSCT (HR: 1.52; *p* = 0.002) [18]. In contrast, in our cohort of newly diagnosed AML patients, PNI did not demonstrate significant discriminatory ability for predicting OS. This discrepancy may be explained by the fact that our study reflects the active leukemia phase at diagnosis, whereas the study by Tang et al. evaluated a more stable clinical period before allogeneic HSCT following remission achievement.

Although the prognostic value of the CONUT score has been demonstrated in some hematological malignancies, evidence regarding its role in AML remains limited [14]. In diffuse large B-cell lymphoma, the CONUT score has been reported as an independent prognostic factor in several studies [21]. In patients with myelodysplastic syndrome receiving decitabine therapy, a higher CONUT score was associated with shorter OS (median OS: 11.0 vs. 17.2 months; *p* = 0.017) [13]. Similarly, in multiple myeloma, a high CONUT score (≥5) was associated with poor OS and identified as an independent prognostic factor (HR = 2.36, *p* = 0.004) [22]. Senjo et al. demonstrated that the conventional CONUT score did not have independent prognostic value in elderly AML patients, whereas the sCONUT score was significantly associated with survival outcomes [14]. Consistent with these findings, the CONUT score did not demonstrate significant prognostic value for OS in our study. However, although the sCONUT score did not show significant discriminatory ability in ROC analysis, it was identified as an independent prognostic factor in both univariate and multivariate Cox regression analyses. This discrepancy may be explained by the fact that ROC analysis evaluates classification performance at a specific time point, whereas Cox regression analysis assesses time-to-event outcomes and mortality risk throughout the follow-up period.

NRI is an objective nutritional index based on serum albumin concentration and body weight and has been used for prognostic evaluation in various cancer types [9,23]. Low NRI has been shown to be independently associated with poor survival in patients with newly diagnosed multiple myeloma [23]. However, the use of NRI in AML populations remains limited, and available data are mainly derived from patients undergoing allogeneic HSCT [11,24]. In these studies, low NRI values were associated with poorer OS (HR: 0.97; *p* < 0.001), although the prognostic discriminatory ability of pre-transplant NRI assessment was limited (C-statistic = 0.59) [11]. In our study, NRI was identified as an independent predictor of mortality after adjustment for age and ELN risk classification. It is well established that both disease-specific biological characteristics and patient-related nutritional status independently influence prognosis in AML [5]. Our study demonstrated that even patients classified within the favorable-risk group according to ELN risk stratification could be further stratified into different risk categories based on NRI scores. These findings suggest that NRI may provide additional prognostic information beyond current AML risk classifications and may contribute to improving risk stratification.

Our study has several limitations. First, the limited sample size, retrospective design, and single-center nature represent important methodological limitations. Second, treatment intensity is an important prognostic factor that may influence survival outcomes in AML and may also be associated with patients’ clinical characteristics and nutritional status. However, the effect of treatment intensity was not evaluated in multivariable analyses in this study, and this should be considered when interpreting the results. Third, temporal changes in nutritional indices during the treatment course were not assessed; such dynamic changes may provide additional prognostic information in future studies. Fourth, our cohort consisted of a heterogeneous patient population with various comorbidities. Although the impact of comorbidities on nutritional status is recognized, all factors that may contribute to malnutrition were not evaluated in detail.

## 5. Conclusions

This study demonstrates that the NRI can serve as an independent prognostic factor for overall survival in newly diagnosed AML patients. As an easily applicable and objective nutritional indicator in clinical practice, NRI can contribute to prognostic assessment. In particular, the co-evaluation of NRI with the ELN risk classification may offer a more holistic prognostic approach that simultaneously reflects both the biological aggressiveness of the disease and the physiological reserve of the patient. Our findings should be validated in larger, prospective, multicenter studies to further establish the clinical value of the NRI for risk stratification in patients with AML.

## Figures and Tables

**Table 1:** ELN 2022 genetic risk classification of AML according to cytogenetic and molecular abnormalities.

Risk Category	Genetic Abnormalities
Favorable	t(8;21)(q22;q22.1)/RUNX1::RUNX1T1 inv(16)(p13.1q22) or t(16;16)(p13.1;q22)/CBFB::MYH11 Mutated NPM1 without FLT3-ITD bZIP in-frame mutated CEBPA
Intermediate	Mutated NPM1 with FLT3-ITD Wild-type NPM1 with FLT3-ITD without adverse-risk genetic abnormalities t(9;11)(p21.3;q23.3)/MLLT3::KMT2A Cytogenetic and/or molecular abnormalities not classified as favorable or adverse
Adverse	t(6;9)(p23.3;q34.1)/DEK::NUP214 KMT2A-rearranged AML t(9;22)(q34.1;q11.2)/BCR::ABL1 inv(3)(q21.3q26.2) or t(3;3)(q21.3;q26.2)/MECOM(EVI1) −5, del(5q), −7, −17/abn(17p) Complex karyotype or monosomal karyotype Mutations in ASXL1, BCOR, EZH2, RUNX1, SF3B1, SRSF2, STAG2, U2AF1, and/or ZRSR2 TP53 mutation

**Table 2:** Scoring systems for the CONUT and Simple CONUT screening tools.

(**A**) CONUT Score				
**Parameters**				
Serum albumin (g/dL)	≥3.5	3.0–3.4	2.5–2.9	<2.4
Score	0	2	4	6
Total cholesterol (mg/dL)	≥180	140–179	100–139	<99
Score	0	1	2	3
Lymphocyte count (cells/μL)	≥1600	1200–1599	800–1199	<799
Score	0	1	2	3
Total Score	0–1	2–4	5–8	9–12
Nutritional Status Group	Normal	Mild	Moderate	Severe
(**B**) Simple CONUT Score				
**Parameters**				
Serum albumin (g/dL)	≥3.5	3.0–3.4	2.5–2.9	<2.4
Score	0	2	4	6
Total cholesterol (mg/dL)	≥180	140–179	100–139	<99
Score	0	1	2	3
Total Score	0–1	2–3	4–5	6–9
Nutritional Status Group	Normal	Mild	Moderate	Severe

**Table 3:** Baseline demographic and clinical characteristics of the study cohort (*n* = 236).

Variable	Category	*n* (%)
Age (years), Median (Min–Max)		55 (18–85)
Gender	Male	140 (59.3)
	Female	96 (40.7)
Treatment Type	Intensive	149 (63.1)
	Low-intensity	87 (36.9)
Treatment Response	CR	182 (77.1)
	Non-CR	54 (22.9)
ELN Risk Category	Favorable	66 (28.0)
	Intermediate	45 (19.1)
	Adverse	125 (53.0)
Vital Status	Alive	146 (61.9)
	Deceased	90 (38.1)

ELN: European LeukemiaNet; CR: complete remission; Min: minimum; Max: maximum.

**Table 4:** Distribution of anthropometric measurements and laboratory parameters.

Parameter	Mean ± SD	Median (Min–Max)
Height (cm)	167.77 ± 9.22	168 (145–190)
Weight (kg)	76.22 ± 14.63	75 (40–122)
Body mass index (kg/m^2^)	27.13 ± 5.12	26.69 (16.48–44.92)
White blood cell count (×10^9^/L)	36,861.83 ± 57,835.95	10,210 (400–322,500)
Hemoglobin (g/dL)	8.00 ± 1.94	7.9 (2.9–13.5)
Platelet count (×10^9^/L)	66.19 ± 80.70	37 (4–550)
Lymphocyte count (×10^9^/L)	8331.48 ± 19,083.71	2550 (70–222,300)
Serum albumin (g/dL)	3.86 ± 0.55	3.9 (2.3–4.9)
Total cholesterol (mg/dL)	140.43 ± 42.94	138.5 (55–290)

**Table 5:** Nutritional status and index scores of the patients.

Parameter	*n* (%)	Mean ± SD	Median (Min–Max)
CONUT Score		2.92 ± 2.00	3 (0–10)
Normal	58 (24.6)		
Mild malnutrition	131 (55.5)		
Moderate malnutrition	44 (18.6)		
Severe malnutrition	3 (1.3)		
Simple CONUT Score		2.12 ± 1.71	2 (0–7)
Normal	97 (41.1)		
Mild malnutrition	96 (40.7)		
Moderate malnutrition	32 (13.6)		
Severe malnutrition	11 (4.7)		
PNI		80.30 ± 95.17	53.5 (26.8–1151.5)
Normal	179 (75.8)		
Low	57 (24.2)		
NRI		99.80 ± 8.87	100.94 (73.25–116.13)
Normal	126 (53.4)		
Mild risk	24 (10.2)		
Moderate risk	76 (32.2)		
Severe risk	10 (4.2)		

**Table 6:** ROC curve analysis of nutritional indices.

Variable	AUC	95% CI	Cut-Off	Sensitivity	Specificity	*p*
CONUT	0.465	0.388–0.542	—	—	—	0.366
Simple CONUT	0.430	0.353–0.506	—	—	—	0.070
PNI	0.537	0.460–0.614	—	—	—	0.340
NRI	0.637	0.566–0.707	101.70	0.69	0.53	**<0.001**

AUC, Area Under the Curve; CI, Confidence Interval; CONUT, Controlling Nutritional Status; PNI, Prognostic Nutritional Index; NRI, Nutritional Risk Index. Bolded *p*-values: *p* < 0.05.

**Table 7:** Survival outcomes based on the NRI score.

Variables	2-Year OS (%)	Median OS, Months (95% CI)	*p*
NRI-high	67.0	NR	**0.004**
NRI-low	40.1	16.80 (8.66–24.94)	

CI: Confidence Interval; OS: overall survival; NR: not reached; NRI: Nutritional Risk Index. Bolded *p*-values: *p* < 0.05.

**Table 8:** Relationship between the NRI score and OS according to ELN risk groups.

ELN Risk	NRI Group	2-Year OS (%)	Median OS, Months (95% CI)	*p*
Favorable	High	93.5	NR	**0.022**
	Low	59.6	NR	
Intermediate	High	52.7	NR	0.620
	Low	41.5	12.43 (0.67–24.19)	
Adverse	High	54.9	17.43 (0.90–33.96)	0.182
	Low	29.9	10.33 (6.43–14.23)	

CI: Confidence Interval; OS: overall survival; NR: not reached (statistical significance evaluated by Kaplan–Meier log-rank test). Bolded *p*-values: *p* < 0.05.

**Table 9:** Univariate Cox regression analysis for overall survival.

Variable	Univariate HR (95% CI)	*p*
ELN Risk Category		
Intermediate vs. Favorable	2.768 (1.374–5.579)	**0.004**
Adverse vs. Favorable	3.758 (2.042–6.916)	**<0.001**
Age	1.023 (1.010–1.036)	**<0.001**
Simple CONUT	1.143 (1.018–1.284)	**0.023**
NRI	0.960 (0.937–0.983)	**0.001**

HR: Hazard Ratio; CI: Confidence Interval; ELN: European LeukemiaNet; CONUT: Controlling Nutritional Status; NRI: Nutritional Risk Index. Bolded *p*-values: *p* < 0.05.

**Table 10:** Multivariate Cox regression analyses for overall survival (two separate models).

Variable	Model A (Simple CONUT) HR (95% CI)	*p*	Model B (NRI) HR (95% CI)	*p*
ELN Risk Category				
Intermediate vs. Favorable	2.265 (1.104–4.652)	0.026	2.101 (1.015–4.349)	**0.046**
Adverse vs. Favorable	3.077 (1.641–5.771)	<0.001	2.851 (1.513–5.371)	**0.001**
Age	1.016 (1.003–1.029)	0.015	1.015 (1.002–1.028)	**0.020**
Simple CONUT	1.135 (1.018–1.284)	0.035	—	—
NRI	—	—	0.967 (0.942–0.992)	**0.009**

Note: Model A adjusted for ELN risk category, age, and Simple CONUT score. Model B adjusted for ELN risk category, age, and NRI score. HR: Hazard Ratio; CI: Confidence Interval; ELN: European LeukemiaNet; CONUT: Controlling Nutritional Status; NRI: Nutritional Risk Index. Bolded *p*-values: *p* < 0.05.

## Data Availability

The data supporting the findings of this study are available from the corresponding author upon reasonable request. However, due to patient confidentiality and privacy regulations, the data are not publicly available and can only be shared when necessary and under appropriate ethical and institutional approvals.

## Supplementary Materials

The following supporting information can be downloaded at: https://www.mdpi.com/article/10.3390/jcm15166179/s1, Table S1. Number of patients at risk at each time point for the Kaplan–Meier overall survival curves.
