# Supplementary material for: Comparative Prognostic Performance of Nutritional Indices in Acute Myeloid Leukemia
Source: J Clin Med. 2026 Aug 10;15(16):6179. doi: 10.3390/jcm15166179 (PMC13512976; doi:10.3390/jcm15166179)
Supplement: Supplementary file 1 [file jcm-15-06179-s001.zip › jcm-4418320-supplementary.pdf]

Supplementary Table S1. Number of patients at risk at each time point for the Kaplan–Meier overall survival curves.

**Subgroup**	**0 months**	**12 months**	**24 months**	**36 months**	**48 months**
Figure 2A. ELN 2022 Risk Stratification					
Favorable risk (n = 66)	66	36	18	9	2
Intermediate risk (n = 45)	45	22	6	1	0
Adverse risk (n = 123)	123	24	11	4	1
Figure 2B. NRI grouping					
High NRI (≥101.70) (n = 105)	105	40	19	8	0
Low NRI (<101.70) (n = 129)	129	42	16	6	3
Figure 3. NRI-stratified OS according to ELN 2022 risk groups					
Favorable risk + High NRI (n = 36)	36	20	10	5	0
Favorable risk + Low NRI (n = 30)	30	16	8	4	2
Intermediate risk + High NRI (n = 21)	21	11	4	1	0
Intermediate risk + Low NRI (n = 24)	24	11	2	0	0
Adverse risk + High NRI (n = 48)	48	9	5	2	0
Adverse risk + Low NRI (n = 75)	75	15	6	2	1

OS, overall survival; NRI, Nutritional Risk Index; ELN, European LeukemiaNet.
